# Role of Hospital Exemption in Europe: position paper from the Spanish Advanced Therapy Network (TERAV)

**DOI:** 10.1038/s41409-023-01962-0

**Published:** 2023-03-25

**Authors:** Fermín Sánchez-Guijo, Cristina Avendaño-Solá, Lina Badimón, Juan A. Bueren, Josep M. Canals, Joaquim Delgadillo, Julio Delgado, Cristina Eguizábal, María-Eugenia Fernández-Santos, Damián García-Olmo, Gloria González-Aseguinolaza, Manel Juan, Francisco Martín, Rosario Mata, Nuria Montserrat, Antonio Pérez-Martínez, José A. Pérez-Simón, Felipe Prósper, Álvaro Urbano-Ispizua, Agustín G. Zapata, Anna Sureda, José M. Moraleda

**Affiliations:** 1 University of Salamanca, IBSAL-University Hospital of Salamanca, Salamanca, Spain; 2 Clinical Pharmacology Department, Hospital Universitario Puerta de Hierro-Majadahonda, IDIPHISA, Madrid, Spain; 3 Department of Molecular Pathology and Therapeutics, IR -Hospital de la Santa Creu i Sant Pau, IIBSantPau, CiberCV, Barcelona, Spain; 4 Biomedical Innovation Unit, CIEMAT, CIBER Rare Diseases and IIS Fundación Jiménez Díaz, Madrid, Spain; 5 Creatio - Production and Validation Center of Advanced Therapies; and Laboratory of Stem Cells and Regenerative Medicine, Department of Biomedical Sciences, Faculty of Medicine and Health Sciences, Institute of Neurosciences, University of Barcelona, Barcelona, Spain; 6 August Pi i Sunyer Biomedical Research Institute (FRCB-IDIBAPS), Barcelona, Spain; 7 Banc de Sang i Teixits, Barcelona, Spain; 8 Hospital Clínic de Barcelona, IDIBAPS, University of Barcelona, Barcelona, Spain; 9 Cell Therapy, Stem Cells and Tissues Group, Basque Center for Blood Transfusion and Human Tissues, Biocruces Bizkaia Health Research Institute, Galdakao, Spain; 10 ATMPs Production Unit. IiS-Gregorio Marañón Health (IiSGM), Gregorio Marañón General University Hospital, Madrid, Spain; 11 “Fundación Jiménez Díaz” University Hospital. Universidad Autónoma de Madrid, Madrid, Spain; 12 Universidad de Navarra, Centro de Investigación Médica Aplicada (CIMA), Programa de Terapia Génica y Regulación de la expresión Génica, Pamplona, Spain; 13 Gene & Cell Therapy Group. GENYO, Centre for Genomics and Oncological Research: Pfizer/University of Granada/Andalusian Regional Government, Granada, Spain; 14 Dpt. de Bioquimica y Biologia Molecular III e Inmunologia. Facultad de Medicina, Universidad de Granada, Granada, Spain; 15 Coordination Unit of the Andalusian Network for the Design and Translation of Advanced Therapies, Seville, Spain; 16 Pluripotency for Organ Regeneration, Institute for Bioengineering of Catalonia (IBEC), The Barcelona Institute of Science and Technology (BIST), Barcelona, Spain; 17 Catalan Institution for Research and Advanced Studies (ICREA), Barcelona, Spain; 18 Universidad Autónoma de Madrid, Institute for Health Research (IdiPAZ), University Hospital La Paz, Madrid, Spain; 19 Department of Hematology, University Hospital Virgen del Rocio, Instituto de Biomedicina de Sevilla (IBIS)/CISC, Universidad de Sevilla, Sevilla, Spain; 20 Servicio de Hematologia y Terapia Celular, Clinica Universidad de Navarra, IdISNA, CIBERONC and CCUN, Pamplona, Spain; 21 Faculty of Biology. Dept of Cell Biology, Complutense University, Madrid, Spain; 22 Departament d’hematologia, Institut Català d’Oncologia-Hospitalet, Institut d’Investigació Biomèdica de Bellvitge (IDIBELL), Universitat de Barcelona, Barcelona, Spain; 23 University of Murcia, IMIB—University Hospital Virgen de la Arrixaca, Murcia, Spain

**Keywords:** Stem-cell research, Translational research

**TO THE EDITOR:**

The Hospital Exemption rule is considered a valuable European legislative initiative that guarantees patient access to novel therapies based on the use of advanced therapy medicinal products (ATMPs) in the Member States. On the occasion of the update of the European General Pharmaceutical Legislation, the Spanish Network of Advanced Therapies (TERAV, formerly TerCel), which has played a recognized leadership in Europe in the development of both academic and pharmaceutically commercialized ATMPs [1], would like to make the following position statement:

The Hospital Exemption Clause was established in the European ATMP regulation EC1394/2007 (Article 28) [2], to enable manufacturing of ATMPs outside the standard centralized marketing authorization common pathway, with the following conditions: *“ATMP is prepared on a non-routine basis according to specific quality standards, and used within the same Member State in a hospital under the exclusive professional responsibility of a medical practitioner, in order to comply with an individual medical prescription for a custom-made product for an individual patient*” and “*Manufacturing of these products shall be authorized by the competent authority of the Member State. Member States shall ensure that national traceability and pharmacovigilance requirements as well as the specific quality standards at Community level in respect of advanced therapy medicinal products for which authorization is required pursuant to Regulation*”.

It is relevant to stress that products administered throughout Hospital Exemption are under the oversight of the corresponding national competent authority of each Member State after careful risk-benefit analysis of the medicinal product. This includes for instance, in the “*Spanish Model”*, a thorough evaluation by the Spanish Agency of Medicines and Medical Devices (AEMPS) of preclinical and clinical data, as well as the inspection and accreditation of the GMP Cell Production Facility and a Pharmacovigilance Plan, among other aspects, which are similar to those required for a centralized marketing authorization, in order to ensure the quality, safety and efficacy of the pharmaceutical product. An unsolved potential problem that the updated regulation should define is the lack of harmonization in the requirements established for Hospital Exemption in the different Member States [3]. On the basis of our very positive experience, we strongly suggest to extend this *Spanish Model* to those systems where preclinical and clinical data or other more stringent criteria are not evaluated for product approval.

In this setting, is important to note that we strongly support the establishment of a public EU registry of the uses and characteristics of ATMPs available through Hospital Exemption. In addition, patients treated with ATMP through Hospital Exemption should be followed up in registries collecting health outcomes, such as those recommended by the EMA and lead by EU based scientific societies. This would be essential for ensuring not only transparency on the safety and availability of these products, but also to generate scientific knowledge and increase the efficiency of the Health Systems and the awareness of all the stakeholders interested, including academia, pharmaceutical industry, regulators, and especially patients’ associations.

As expected, the number of ATMPs approved by Hospital Exemption is very limited in the EU, and these products have been mainly developed to provide treatments for patients not included or ineligible for participating in clinical trials or where ATMPs were not considered suitable for commercial development. Hospital Exemption is therefore an essential tool to ensure timely access to safe, effective, and legally regulated treatments for patients with rare diseases or those lacking effective treatments or better therapeutic alternatives [4]. Therefore, Hospital Exemption is a stimulus to promote innovation in advanced therapies in European academic institutions. As commented before, the most positive consequence is the increased access of advanced therapies to our patients. The main reasons for this are, amongst others, the fact that they may cover indications not yet approved for commercial CARTs and other ATMPs, the expedited manufacturing time (and at a lower cost) of an academic product, and that they may provide treatment for diseases where pharmaceutical companies are not focused on. Besides, Hospital Exemption clause alleviates the worrying difficulties of supplies to European countries of ATMPs manufactured outside the EU.

Moreover, it is very important to highlight our support of the use of the exemption clause with academic ATMPs also for indications in which there is a commercial product approved, as in the case for instance of CAR-T in hematological malignancies, since many patients do not have access, for a number of reasons, to the approved commercial products. Moreover, these developments may provide important technical and manufacturing improvements that can lead to greater efficacy, lower toxicity or better access. In this regard, we are looking forward to the successes of the EMA pilot program recently launched to help academic and non-profit developers navigate the regulatory processes and optimize development of ATMPs within the EU [5, 6].

In our opinion, the centralized approval and the Hospital Exemption are more complementary than competitive approaches, and Hospital Exemption represents a unique opportunity of partnership between academia and pharmaceutical industry in the benefit of patients, and is one of the most important assets in the European Union to get these highly innovative and disruptive therapies accessible to our national health systems.
